# Multiyear environmental surveillance in a pediatric teaching hospital: association between airborne mold spores and invasive mold infections

**DOI:** 10.1017/ice.2025.10264

**Published:** 2025-11

**Authors:** Bethany Phillips, Zachary M. Most, Bryan Connors, Patricia Jackson, Michael E. Sebert

**Affiliations:** 1 Infection Prevention and Control, Children’s Health System of Texas, Dallas, TX, USA; 2 Division of Infectious Diseases, Department of Pediatrics, University of Texas Southwestern Medical Center, Dallas, TX, USA; 3 Environmental Health & Engineering, Newton, MA, USA; 4 Infection Prevention, Scottish Rite for Children, Dallas, TX, USA

## Abstract

**Background:**

The utility of routine environmental sampling to monitor the airborne fungal load (AFL) in healthcare settings is uncertain.

**Methods:**

AFL was measured by monthly cultures at a tertiary-care pediatric hospital from November 2018 through October 2023 on eleven units caring for patients at risk for invasive mold infection (IMI). Surveillance for healthcare-associated IMI was conducted for all patients in the healthcare system using locally developed definitions for possible, probable, and definite hospital-onset infections. Poisson regression was used to analyze the association between AFL and monthly IMI rates.

**Results:**

78 cases of IMI were identified during the period of AFL monitoring. Of these, 51 infections were classified as healthcare-associated probable or proven IMI and were tested for association with AFL measurements. There was not a significant facility-wide association between the average monthly AFL and the overall IMI rate. On units where hematology/oncology patients were treated, however, an increase in average monthly local AFL for opportunistic fungal pathogens of 1 CFU/m^3^ was associated with a 1.48-fold increase in the IMI rate for these patients (95% CI 1.00–2.19, *P* = .05). The AFL for *Aspergillus* species on these units showed a particularly strong association with the hematology/oncology IMI rate (15.9-fold elevation for an increase of 1 CFU/m^3^ [95% CI 2.8–90.7, *P* = .002]). Neither hematology/oncology nor facility-wide IMI rates showed comparable associations with changes of the AFL in outdoor air.

**Conclusions:**

Regular monitoring of AFL on targeted hospital units may identify periods when hematology/oncology patients are at increased risk for IMI.

## Introduction

Invasive mold infections (IMI) cause substantial morbidity and mortality among patients hospitalized with hematologic malignancies and stem cell transplant (SCT) recipients yet also impact other individuals including surgical patients.^1–6^ Environmental controls including high-efficiency particulate air (HEPA)-filtration are important for limiting exposure to airborne mold spores among vulnerable patients in healthcare settings.^7,8^ Sampling for airborne fungi has been utilized as a supplement to environmental controls during periods of major construction activity or outbreaks.^7,9^ The utility of such environmental sampling during other times of routine hospital operations, however, is unclear due to several limitations. Thresholds beyond which elevated levels of airborne mold spores increase IMI risk are unknown,^7,8,10^ and transient spore bursts may not be captured by periodic testing.^11,12^ In addition, it is challenging to determine whether individual IMI cases should be attributed to healthcare exposures given the absence of standardized surveillance definitions and uncertain incubation periods.^13^ Consequently, practices regarding measurement of airborne fungal loads (AFL) and surveillance for IMI vary widely among infection prevention (IP) programs at U.S. hospitals.^14^

Since late 2018, our hospital has conducted routine monthly sampling to monitor the viable AFL on units with the most vulnerable patients. Testing was started in preparation for an extended maintenance project on the ventilation system requiring intermittent closures of clinical areas from June 2019 until August 2020. Following that work, regular air sampling was continued due to perceived benefits. Additionally, our IP department has conducted standardized facility-wide surveillance for IMI since before starting routine air sampling. We now report this experience with AFL monitoring and IMI surveillance.

## Methods

### Study setting

We conducted a retrospective ecological study of AFL in the healthcare environment and IMI cases at a 490-bed tertiary-care pediatric teaching hospital in Texas, USA. The hospital includes eleven units identified as caring for patients at highest risk for IMI: two pediatric intensive care units, one cardiac intensive care unit (CICU) with an adjoining step-down unit, three neonatal intensive care units (NICU), and a hematology/oncology program with four inpatient units including a positive-pressure SCT unit. Ventilation for these units is provided by air-handling units equipped with central HEPA-filtration.

### AFL measurement

Regular air sampling for fungal cultures was conducted approximately monthly as detailed in the Supplemental Materials. Average AFL values were calculated by unit and facility-wide for each collection date. Because sampling dates were irregularly spaced, average monthly AFL was estimated by linear interpolation to generate daily AFL values for dates between measurements and then averaging daily values over the calendar month.

### IP program responses to AFL findings

The initial framework used for interpreting and managing AFL results considered both total and opportunistic fungal pathogen levels. Each unit was assigned into an action response category based on these values and the thresholds in Table 1.

**Table 1.** Action categories for IP management of AFL results during the study period

Action response category	Total AFL (CFU/m^3^)	Opportunistic fungal pathogen AFL (CFU/m^3^)	Unit evaluation steps
Green	0–4	< 1	None
Yellow	5–10	1–5	Visual inspection for water damage, dust or outside air intrusion; evaluation of local construction projects; review of maintenance issues including HVAC system and plumbing leaks
Red	> 10	> 5	As above for yellow, plus: Visual inspection of HVAC air handling unit; repeat air sampling for fungal cultures after evaluation and remediation

AFL, airborne fungal load; CFU, colony forming units. Notes. These categories and thresholds applied specifically to AFL measurements obtained on HEPA-filtered units housing patients at high risk for invasive mold infection. The framework for characterizing the air sampling data described above was initially developed to guide the priority and type of follow-up actions during and specific to the HVAC system maintenance project. The same framework was later employed to guide routine IP responses to continued AFL surveillance in the absence of other established standards.

### IMI surveillance

We reviewed IMI surveillance data for January 2018 to December 2023. The IP department conducted facility-wide surveillance for IMI as described in the Supplemental Materials. Cases were classified as proven, probable, or possible IMI based on published criteria.^15^ After these criteria were updated in 2020, IMI surveillance from earlier years was reviewed for agreement with the revised definitions. Cases were furthermore classified as definite, probable, or possible hospital-onset (HO) or community-onset (CO) based on timing of sign/symptom onset using criteria in Table 2. Infections with *Candida* species and other yeast-like fungi such as *Trichosporon* were excluded from surveillance. Endemic mycoses were retained within the surveillance, but all cases of endemic mycoses were classified as CO since reactivation can occur months or years after exposure.^16^

**Table 2.** Surveillance definitions for IMI onset classification

Onset class	IMI surveillance definition
Definite HO	Sign/symptom onset after HD 28, or IMI that develops at the site of a medical device or surgical incision that was not present prior to admission
Probable HO	Sign/symptom onset HD 15 to 28
Possible HO	Sign/symptom onset HD 8 to 14, or onset before HD 8 (including prior to admission) with other significant healthcare facility exposure^*^
CO^†^	Sign/symptom onset before HD 8 and no other significant healthcare facility exposure^*^

IMI, invasive mold infection; HO, hospital-onset; CO, community-onset; HD, hospital day

* Other significant healthcare facility exposure was defined as ≥ 1 hospitalization or ≥ 2 outpatient visits during the month prior to sign/symptom onset.

† Endemic mycoses including infections with *Histoplasma* or *Coccidioides* were always considered to be CO in keeping with National Healthcare Safety Network guidance,^22^ but otherwise the same definitions applied to all mold species.

### Statistical analysis

Categorical variables were expressed as counts or percentages, whereas continuous variables were described using means, medians, and percentile ranges. Fisher’s exact test was used to compare proportions, and continuous variables were analyzed using the Mann-Whitney U test. Poisson regression with an offset of ln(10,000s of patient days) was used to analyze the association between monthly AFL and IMI rates, for interrupted time series analyses, and to calculate IMI rate ratios. AFL values were also categorized and the chi-square test for trend was used to assess the association with IMI rates. Poisson regression was conducted using SAS Enterprise Guide version 7.15 (Cary, NC, USA). Other statistical analyses were performed using GraphPad Prism version 10.2.3 (San Diego, CA, USA). A two-sided alpha threshold of 0.05 was used to assess statistical significance.

### Human subjects

The Human Research Protection Program at the University of Texas Southwestern Medical Center, which has oversight of research at all facilities within our health system, determined that this project did not meet the definition of human subjects research.

## Results

### AFL measurements

**Figure 1.**
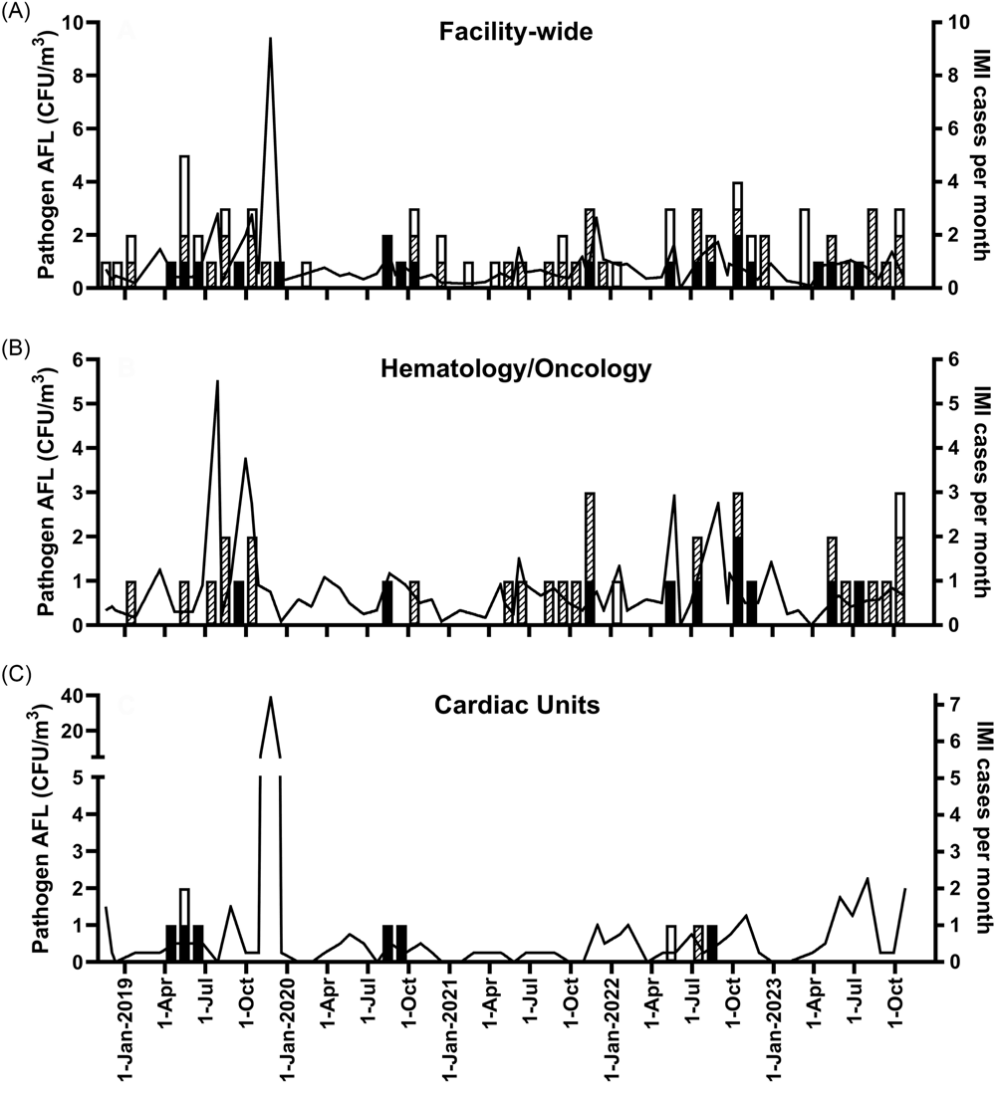
AFL and IMI by month for (A) facility-wide measurements, (B) hematology/oncology, and (C) cardiac units. Solid lines show AFL of opportunistic fungal pathogens. Bars indicate numbers of probable or proven IMI cases per month that were classified as probable or definite HO (solid black bars), possible HO (diagonal stripes), or community-onset (open white bars). AFL, airborne fungal load; IMI, invasive mold infection; HO, hospital-onset.

Concentrations of airborne fungi measured in inpatient units, operating rooms, and outdoor air samples are shown in Table 3. The median total AFL for occupied inpatient units (2 CFU/m^3^) was lower than in outdoor air (48 CFU/m^3^, *P* < .0001) but higher than in the operating rooms (0 CFU/m^3^, *P* < .0001). The same pattern was seen for the AFL of opportunistic fungal pathogens. Most colonies of airborne fungi in outdoor samples were *Cladosporium* spp., which were also common among isolates from inpatient units. The most frequent airborne fungal pathogens identified from inpatient units were *Penicillium* spp., followed by dematiaceous molds and *Aspergillus* spp. AFL measurements fluctuated sharply over even short intervals (Figure 1) but seasonal patterns were not evident. Histograms showing complete frequency distributions of AFL measurements are in Supplemental Figure S1. Monthly AFL values for fungal pathogens were only slightly higher during the ventilation system maintenance project than during the remainder of the study period for facility-wide measurements (median 0.79 [IQR 0.53–1.93] vs 0.59 [IQR 0.42–0.91] CFU/m^3^, *P* = .07) and for units caring for hematology/oncology patients (median 0.79 [IQR 0.44–1.09] vs 0.54 [IQR 0.36–0.78] CFU/m^3^, *P* = .11).

**Table 3.** Abundances of fungi identified in surveillance air cultures

Fungal types identified	Inpatient units (CFU/m^3^, *N* = 1405)				Operating rooms (CFU/m^3^, *N* = 136)				Outdoor air samples (CFU/m^3^, *N* = 77)			
		Percentiles				Percentiles				Percentiles		
	Mean	50^th^	75^th^	95^th^	Mean	50^th^	75^th^	95^th^	Mean	50^th^	75^th^	95^th^
All fungal species combined	2.67	2	3	8	0.41	0	1	2	61.9	48	75	135
Opportunistic fungal pathogens	0.85	0	1	3	0.10	0	0	1	7.4	6	11	19
*Aspergillus* spp.	0.15	0	0	1	0.04	0	0	0	2.44	1	4	9
*Penicillium* spp.	0.45	0	0	1	0.01	0	0	0	2.91	1	4	12
Dematiaceous molds	0.20	0	0	1	0.04	0	0	0	0.51	0	0	4
Mucorales^*^	0.02	0	0	0	0.00	0	0	0	0.26	0	1	1
*Fusarium* spp.	0.01	0	0	0	0.00	0	0	0	1.18	0	1	8
*Cladosporium* spp.	0.73	0	1	3	0.10	0	0	1	50.9	36	65	133
Yeast^†^	0.11	0	0	1	0.03	0	0	0	0.21	0	0	0
Non-sporulating fungal isolates	0.91	0	1	3	0.18	0	0	1	3.26	2	5	12
Other fungal species^‡^	0.10	0	0	1	0.01	0	0	0	0.23	0	0	2

* For Mucorales, colony counting may underestimate the number of these organisms present in a specimen due to the predilection for rapidly spreading across plates. For comparison, Supplemental Table S3 gives percentages of cultures positive at any level for each category of fungi and confirms that Mucorales isolates were rare in indoor samples.

† Includes yeast, which were not further identified, and yeast-like fungi such as *Geotrichum* spp.

‡ Other fungal species represent infrequently identified organisms, including some considered opportunistic pathogens and others categorized as non-pathogens as described in Supplemental Materials.

Notes. Data presented are descriptive statistics for all individual surveillance air cultures from the given areas. All inpatient units sampled were equipped with HEPA-filtration.

### IMI case surveillance

From January 2018 until December 2023, 96 cases of possible, probable, or proven IMI were identified (Table 4) of which 78 occurred during the period of AFL monitoring. Time to sign/symptom onset ranged from before admission to HD (hospital day) 151. Most cases were on the hematology/oncology service (including SCT recipients), but 13 cases occurred in cardiology patients, including 8 infections classified as probable or definite HO. Among cardiology patients, all HO IMI cases occurred in post-surgical or heart transplant patients. IMI cases resulted from a wide range of pathogens, with *Aspergillus* identified from only 32% of infections (Table 4). Hematology/oncology cases predominately affected the respiratory tract with pulmonary, pleural, or sinonasal infections, whereas those in the cardiac population frequently involved the mediastinum, sternum, or heart (Table S1). Although patients on all other services collectively accounted for 34 IMI cases, most of these infections qualified as CO with very few being probable or definite HO (Table 4). Unlike the hematology/oncology or cardiology cases, a substantial number of these other cases were evaluated by clinicians as likely representing a contaminated culture or fungal colonization rather than invasive infection (Table S1). We therefore focused particularly on the hematology/oncology and cardiology populations during subsequent analyses aimed at understanding healthcare-associated infections.

**Table 4.** Characteristics of patients with IMI by onset class

IMI case characteristics	All IMI cases (*n* = 96)	Definite HO (*n* = 15)	Probable HO (*n* = 10)	Possible HO (*n* = 43)	CO (*n* = 28)
Age, years (median, IQR)	11.1 (6.2–15.2)	10.0 (1.9–16.0)	10.3 (1.4–15.4)	9.0 (5.1–14.8)	14.1 (8.9–16.7)
Clinical service (%)					
Hematology/Oncology	49 (51)	6 (40)	8 (80)	33 (77)	2 (7)
Cardiac units	13 (14)	7 (47)	1 (10)	2 (5)	3 (10)
Other	34 (35)	2 (13)	1 (10)	8 (19)	23 (82)
HD of sign/symptom onset (median, IQR)	1 (<1 to 14)	48 (30 to 59)	18.5 (16 to 21.25)	1 (<1 to 9)	<1 (<1 to <1)
IMI category (%)					
Proven IMI	74 (77)	13 (87)	10 (100)	28 (65)	23 (82)
Probable IMI	15 (16)	1 (7)	0	9 (21)	5 (18)
Possible IMI	7 (7)	1 (7)	0	6 (14)	0
Infection site(s) (%)					
Pulmonary or pleural	35 (36)	4 (27)	2 (20)	20 (47)	9 (32)
Sinonasal	20 (21)	3 (20)	4 (40)	13 (30)	0
Skin or soft tissue	20 (21)	3 (20)	2 (20)	10 (23)	5 (18)
Mediastinum or sternum	7 (7)	6 (40)	1 (10)	0	0
Bloodstream or disseminated	6 (6)	0	1 (10)	2 (5)	3 (11)
Musculoskeletal	6 (6)	1 (7)	0	0	5 (18)
Abdomen or pelvis	4 (4)	0	1 (10)	2 (5)	1 (4)
Lymph node	3 (3)	0	0	0	3 (11)
Central nervous system	3 (3)	0	0	2 (5)	1 (4)
Endocarditis	2 (2)	0	0	0	2 (7)
Eye	1 (1)	0	0	1 (2)	0
Clinically considered contaminant or colonization rather than infection (%)	13 (14)	0	0	3 (7)	10 (36)
Fungal pathogen(s) (%)					
Hyaline molds					
*Aspergillus* ^*^	31 (32)	4 (27)	3 (30)	18 (42)	6 (21)
Positive AGM only	5 (5)	1 (7)	0	4 (9)	0
*Fusarium*	9 (9)	0	2 (20)	5 (12)	2 (7)
*Penicillium*	4 (4)	0	0	1 (2)	3 (11)
*Paecilomyces*	1 (1)	0	0	0	1 (4)
*Trichoderma*	1 (1)	0	0	0	1 (4)
Hyaline mold, NOS	3^†^ (3)	0	0	1^†^ (2)	2 (7)
Dematiaceous molds					
*Bipolaris*/*Curvularia*	21 (22)	6 (40)	2 (20)	12 (28)	1 (4)
*Alternaria*	5 (5)	1 (7)	2 (20)	2 (5)	0
*Exserohilum*	4 (4)	0	0	2 (5)	2 (7)
*Exophiala*	2 (2)	0	0	1 (2)	1 (4)
*Cladosporium*	4 (4)	0	0	2 (5)	2 (7)
Dematiaceous mold, NOS	3 (3)	0	0	2 (5)	1 (4)
Mucorales^‡^					
*Rhizopus*	6 (6)	1 (7)	2 (20)	2 (5)	1 (4)
*Rhizomucor*	2 (2)	0	1 (10)	0	1 (4)
*Mucor*	2 (2)	2 (13)	0	0	0
Zygomycetes, NOS	2 (2)	1 (7)	0	1 (2)	0
Dimorphic fungi					
*Histoplasma*	4 (4)	0	0	0	4 (14)
*Coccidioides*	1 (1)	0	0	0	1 (4)
*Trichophyton*	2 (2)	0	0	1 (2)	1 (4)
*Nannizziopsis*	1 (4)	0	0	0	1 (4)
No fungal pathogen identified	7 (7)	1 (7)	0	6 (14)	0
Multiple mold species^§^	15 (16)	1 (7)	2 (20)	9 (21)	3 (11)

IMI, invasive mold infection; HO, hospital-onset; CO, community-onset; IQR, interquartile range; HD, hospital day; AGM, *Aspergillus* galactomannan; NOS, not otherwise specified.

* Including cases with positive AGM testing only.

† Including one isolate identified as *Penicillium*/*Paecilomyces*.

‡ Including isolates identified only as Zygomycetes, which could represent species other than Mucorales.

§ Isolates from cases with multiple mold species are also listed separately with the individual molds identified.

### Monthly average AFL and IMI rates

To examine potential relationships between AFL measurements and healthcare-associated IMI, we limited analyses to cases of proven or probable IMI. Reflecting our surveillance process that relied largely on reports of fungal culture results and histopathology findings, these IMI cases represented 93% of the total infections identified. Overall, there were 51 cases of probable or proven IMI that were considered potentially healthcare-associated (i.e., possible, probable, or definite HO) during the AFL monitoring period. These HO categories were combined for subsequent analyses unless otherwise noted.

**Figure 2.**
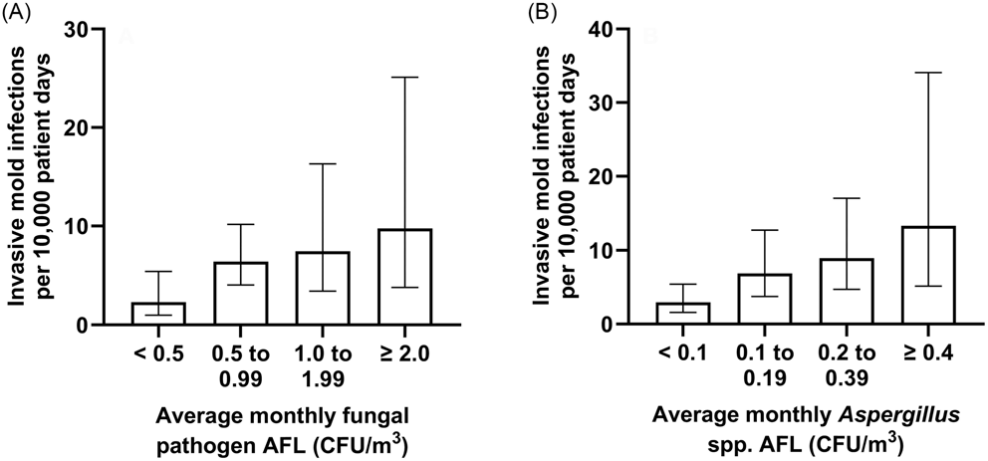
Rates of invasive mold infections among hematology/oncology patients during months when the average AFL on units where these patients received care was in the indicated ranges for (A) opportunistic fungal pathogens, and (B) *Aspergillus* species. Chi-square test for trend for panel A, *P* = .02; and for panel B, *P* = .001. Bars indicate average rates and error bars show 95% confidence intervals. AFL, airborne fungal load; CFU, colony forming units.

There was not a significant association between the average monthly AFL and the IMI rate when examined at the facility-wide level (Table 5). For hematology/oncology patients, an increase of 1 CFU/m^3^ in the average monthly AFL for opportunistic fungal pathogens on the units where these patients receive care was associated with a 1.48-fold (95% CI 1.00–2.19, *P* = .05) increase in the IMI rate. In contrast, there was not a significant change for cardiac patients. The hematology/oncology IMI rate ranged from an average of 2.3 cases per 10,000 patient days (95% CI 1.0–5.4) during months when the local unit pathogen average AFL was less than 0.5 CFU/m^3^ up to an average of 9.8 cases per 10,000 patient days (95% CI 3.8–25.1) when the average pathogen AFL was 2.0 CFU/m^3^ or greater (Figure 2A, chi-square test for trend *P* = .02). As a control for the temporal relationship between AFL and IMI events, we found that there was not a significant association between the hematology/oncology IMI rate for the prior month and the local unit AFL for the following month (1.36-fold change for an increase of 1 CFU/m^3^ [95% CI 0.89–2.10, *P* = .16]).

**Table 5.** IMI rates and monthly opportunistic fungal pathogen AFL

Proven and probable IMI cases per 10,000 patient days for month of AFL measurement	Change in IMI rate per change of 1 CFU/m^3^ in average monthly fungal pathogen AFL					
	Local unit counts		Facility-wide counts		Outdoor counts	
	Fold change return (95% CI)	*P* value	Fold change return (95% CI)	*P* value	Fold change return (95% CI)	*P* value
Definite, probable or possible HO						
Facility-wide rate	N/A		1.19 (0.94–1.51)	.16	0.99 (0.94–1.05)	.75
Hematology/Oncology rate	1.48 (1.00–2.19)	.05	1.11 (0.80–1.54)	.52	0.93 (0.85–1.01)	.08
Cardiac units rate	0.72 (0.15–3.46)	.68	1.00 (0.41–2.43)	.99	1.07 (0.94–1.22)	.33
Definite or probable HO						
Facility-wide rate	N/A		1.13 (0.73–1.73)	.58	1.03 (0.95–1.12)	.46
Hematology/Oncology rate	1.57 (0.80–3.11)	.19	1.15 (0.66–2.01)	.63	0.93 (0.80–1.09)	.35
Cardiac units rate	0.70 (0.12–4.18)	.70	0.97 (0.35–2.67)	.95	1.07 (0.93–1.23)	.37

IMI, invasive mold infection; AFL, airborne fungal load; CFU, colony forming units; HO, hospital onset.

*Notes*. All rates are calculated based on combined numbers of proven and probable IMI cases. Local air pathogen counts for the hematology/oncology population include those taken on inpatient hematology/oncology floors, stem cell transplant unit, and pediatric intensive care units. Local air pathogen counts for the cardiac population were limited to those taken in the CICU as samples were not collected on the acute care cardiology floor.

The types of fungi identified from the air affected the association with IMI (Supplemental Table S2). The local average AFL for *Aspergillus* species showed a strong association with the hematology/oncology IMI rate (15.9-fold elevation for an increase of 1 CFU/m^3^ [95% CI 2.8–90.1, *P* = .002]). Figure 2B shows the increase in this IMI rate with even low levels of average airborne *Aspergillus*. The AFL for pathogens excluding *Aspergillus* also appeared to retain a borderline association with the hematology/oncology IMI rate. This association was strengthened when a combined IMI rate was calculated for the month of the AFL measurement plus the following month (1.42-fold elevation per increase of 1 CFU/m^3^ [95% CI 1.04–1.95, *P* = .03]). When non-pathogens were included, however, no association was seen between total AFL on local units and the hematology/oncology IMI rate.

Because outdoor air quality might influence AFL on inpatient units and exposure before admission could contribute to later development of IMI, we examined whether IMI rates were associated with AFL measured in outdoor air samples. AFL readings in outdoor air, however, did not positively correlate with indoor values (Figure S2). Consistent with this finding, associations between IMI rates and AFL measured outside the hospital were not similar to those seen for indoor AFL values (Table 5 and Supplemental Table S2).

To examine further the relationship between AFL and IMI rates, we conducted sensitivity analyses limiting the IMI rate to definite or probable HO cases (Table 5). The magnitude of association with AFL on local units was similar for hematology/oncology patients, but confidence intervals were wider reflecting the smaller number of cases.

## Discussion

These results provide preliminary evidence for an association between fungal pathogen AFL and the rate of healthcare-associated IMI among pediatric hematology/oncology patients. Our findings contrast with the conclusions of previous investigators^7,11,12^ who have questioned the utility of AFL monitoring during routine operations for predicting invasive aspergillosis (IA) among immunocompromised patients at hospitals with good environmental controls including HEPA-filtration. Other studies have reported associations between AFL and IA but have been limited by conflicting results among different units and observation periods^17^ or very small numbers of IA cases.^18^ Our use of frequent AFL measurements and systematic IMI surveillance with consistent methodologies over an extended period may have facilitated finding an association.

Several factors may also have contributed to the frequency of IMI events and increased the ability of our study to identify this association. Routine evaluation for fungal infection in patients with prolonged febrile neutropenia at our hospital includes nasal endoscopy even in the absence of sinus symptoms. This practice was previously reported to increase identification of fungal infections^19^ and likely contributed to the high frequency of sinonasal infections among oncology patients. In addition, the cardiac service at our hospital manages a high volume of critically ill patients including a large pediatric heart transplant program. Delayed sternal closure and bedside invasive procedures are often required in this population and may increase risk for IMI.

Although extended maintenance on the ventilation system was the impetus for starting regular environmental monitoring, AFL measurements during the maintenance project were only marginally higher than at other times, and infection rates were not appreciably different (Figure S4). Units were closed and barricaded during the work, with their ventilation systems isolated from the rest of the hospital where AFL measurements were taken. The results of this study therefore reflect the impact of AFL surveillance under conditions of essentially routine hospital operations. Median AFL measurements for indoor sampling were generally low, which may reflect the impact of HEPA-filtration for the inpatient units and operating rooms being monitored.

The absence of established mold concentrations above which IMI risk increases has limited the acceptance of routine AFL monitoring, although thresholds for airborne *Aspergillus* ranging from less than 0.1 to 5 CFU/m^3^ have been recommended in areas where patients are at highest risk for IMI and stringent environmental controls are in place.^7,10^ Our analyses now show that an increase in the fungal pathogen AFL of 1 CFU/m^3^ was associated with a 1.48-fold increase in the IMI rate among hematology/oncology patients and that increases in the *Aspergillus* AFL appeared to have an even larger impact. As discussed in the Supplemental Materials, these data may start to inform the selection of relevant action thresholds for future AFL monitoring in healthcare facilities.

The prolonged turnaround time (TAT) of fungal cultures has also been considered a barrier to using the results of such environmental cultures in routine IP activities.^11,12^ Our program utilized a commercial culture protocol with an incubation period of just five days, which generally yielded reports within about a week of collection. Although this short incubation may have contributed to some isolates being identified only as non-sporulating mold, the shorter TAT provided more timely and actionable results. In several instances, AFL monitoring led to interventions intended to protect patients or facilitated IP investigations. Areas of the CICU and NICU were closed on two occasions due to unusually high AFL readings for *Aspergillus* and/or *Penicillium*. Investigation of the CICU revealed a potential mold source in an adjacent non-clinical space that was undergoing renovations, and antifungal post-exposure prophylaxis was provided to high-risk cardiac patients (i.e., those with heart transplants, open sternal incisions, or supported by ventricular assist devices or extracorporeal membrane oxygenation). After the NICU closure, elevated *Penicillium* AFL was unexpectedly found again in the new unit to which patients had been moved. This finding triggered close evaluation of equipment that had been moved, which identified an infant incubator with mold on an internal humidifier filter. No infections were linked to either episode, but we cannot know if infections were prevented by our interventions. On another occasion when two healthcare-associated IMI cases were identified in the CICU, unremarkable data from recent surveillance cultures contributed to expanding the scope of investigation promptly to other areas through which the patients had been transported during care.

Our study has several limitations including that it was conducted at a single center. Distributions of AFL measurements and frequencies of different fungal species may vary at institutions in different geographic regions or with different building infrastructure. Another limitation of our study was the absence of AFL measurements on additional units of the hospital where patients are typically at lower risk for IMI or in public spaces such as lobbies and cafeterias. The AFL sampling regimen was designed based on pragmatic needs and constraints of our IP program rather than as a planned research initiative. Because we conducted a retrospective analysis that involved multiple hypothesis testing, all results should be considered exploratory. The associations found between AFL and IMI rates in hematology/oncology patients, however, are particularly plausible because these findings were specific to local measurements on the units treating these vulnerable patients and among whom infections of the respiratory tract were consistent with inhalational exposure. IMI rates analyzed for this study were normalized to monthly patient-days of hospitalization. We were unable to adjust monthly IMI rates for individual patient-level risk factors such as neutropenia, antifungal prophylaxis, or surgical status, but it seems unlikely that these patient-level factors would have varied systematically with the measured AFL. Mold-active antifungal prophylaxis was routinely provided during the study period to patients with acute myelogenous leukemia, selected acute lymphoblastic leukemia (ALL) patients with T-cell ALL, relapsed or refractory B-cell ALL, or chromosomal disorders such as trisomy 21, and to allogeneic SCT patients within 6 months after transplant.

The absence of standardized definitions for healthcare-associated IMI for which the incubation period remains unknown^13^ contributes to uncertainty about how these events should be classified. We used locally developed HO categories that acknowledged the uncertainty around IMI incubation periods by classifying infections as possible or probable rather than definite HO over broad time spans. These definitions had been developed for surveillance by our IP program prior to undertaking the analyses presented here and were selected in part to avoid overstating the certainty that any particular infection was healthcare-acquired. Our primary analysis of the association between AFL and IMI rates combined data from possible, probable, and definite HO infections, which together were considered as healthcare-associated IMI. This aggregate category included any infections with sign/symptom onset after the first week of hospitalization or with defined healthcare exposures before admission and was thus comparable to definitions selected for prior studies of healthcare-associated IMI.^13,20,21^

Very few infections within the hematology/oncology population were classified as CO because other significant healthcare exposure during the month before sign/symptom onset typically gave a classification of at least a possible HO. The associations between AFL and IMI for hematology/oncology patients—including possible HO events—suggests that at least a portion of the early hospitalization infections classified as possible HO were likely acquired in the healthcare setting. The alternative potential for some IMI to develop after an extended period of latency or incubation, however, is suggested by the fact that the association between hematology/oncology IMI and the non-*Aspergillus* pathogen AFL was strengthened by inclusion of IMI data from the month following the AFL measurement. These observations may contribute to future efforts that are needed to standardize criteria for healthcare-associated IMI.

Within the limitations above, our results may provide useful comparison data for other hospitals performing AFL measurements. Further work is needed to extend these observations to other healthcare facilities and more fully define the role that routine AFL surveillance may play in mitigating the risk of IMI among immunocompromised patients.

## Supporting information

Phillips et al. supplementary material 1 Phillips et al. supplementary material

Phillips et al. supplementary material 2 Phillips et al. supplementary material

## Supplementary material

To view supplementary material for this article, please visit https://doi.org/10.1017/ice.2025.10264

## References

[1] Pana ZD , Roilides E , Warris A , Groll AH , Zaoutis T. Epidemiology of invasive fungal disease in children. *J Pediatric Infect Dis Soc* 2017;6:S3–S11. 10.1093/jpids/pix046 PMC5907880 28927200

[2] Green AM , Otto WR. Fungal infections in children with haematologic malignancies and stem cell transplant recipients. Br J Haematol 2020;189:607–324. 32159231 10.1111/bjh.16452 PMC7231650

[3] Lehrnbecher T , Schöning S , Poyer F , et al. Incidence and outcome of invasive fungal diseases in children with hematological malignancies and/or allogeneic hematopoietic stem cell transplantation: results of a prospective multicenter study. Front Microbiol 2019;10:681. 31040830 10.3389/fmicb.2019.00681 PMC6476895

[4] Zaoutis TE , Heydon K , Chu JH , Walsh TJ , Steinbach WJ . Epidemiology, outcomes, and costs of invasive aspergillosis in immunocompromised children in the United States, 2000. *Pediatrics* 2006;117:e711–716. 10.1542/peds.2005-1161 16533892

[5] Denning DW. Global incidence and mortality of severe fungal disease. *Lancet Infect Dis* 2024;24:e428–e438. 10.1016/S1473-3099(23)00692-8 38224705

[6] Hariri G , Genoud M , Bruckert V , et al. Post-cardiac surgery fungal mediastinitis: clinical features, pathogens and outcome. Crit Care 2023;27:6. 36609390 10.1186/s13054-022-04277-6 PMC9817255

[7] Marek A , Meijer EFJ , Tartari E , et al. Environmental monitoring for filamentous fungal pathogens in hematopoietic cell transplant units. *Med Mycol* 2023;61:myad103. 10.1093/mmy/myad103 37793805

[8] Healthcare Infection Control Practices Advisory Committee. Guidelines for Environmental Infection Control in Health-Care Facilities (2003), Updated: July 2019. Centers for Disease Control and Prevention website. https://www.cdc.gov/infection-control/hcp/environmental-control/index.html. Published 2019. Accessed February 20, 2025.

[9] Kanamori H , Rutala WA , Sickbert-Bennett EE , Weber DJ. Review of fungal outbreaks and infection prevention in healthcare settings during construction and renovation. Clin Infect Dis 2015;61:433–444. 25870328 10.1093/cid/civ297

[10] Morris G , Kokki MH , Anderson K , Richardson MD. Sampling of Aspergillus spores in air. J Hosp Infect 2000;44:81–92. 10662557 10.1053/jhin.1999.0688

[11] Falvey DG , Streifel AJ. Ten-year air sample analysis of Aspergillus prevalence in a university hospital. J Hosp Infect 2007;67:35–41. 17719681 10.1016/j.jhin.2007.06.008

[12] Rupp ME , Iwen PC , Tyner LK , Marion N , Reed E , Anderson JR. Routine sampling of air for fungi does not predict risk of invasive aspergillosis in immunocompromised patients. J Hosp Infect 2008;68:270–271. 18226417 10.1016/j.jhin.2007.11.017

[13] Nicolle MC , Benet T , VanHemes P. Aspergillosis: nosocomial or community-acquired? *Med Mycol* 2011;49:S24–S29. 10.3109/13693786.2010.509335 20818924

[14] Gold JAW , Jackson BR , Glowicz J , Mead KR , Beer KD. Surveillance practices and air-sampling strategies to address healthcare-associated invasive mold infections in Society for Healthcare Epidemiology of America (SHEA) Research Network hospitals—United States, 2020. Infect Control Hosp Epidemiol 2022;43:1708–1711. 34266512 10.1017/ice.2021.285 PMC8761226

[15] Donnelly JP , Chen SC , Kauffman CA , et al. Revision and update of the consensus definitions of invasive fungal disease from the European Organization for Research and Treatment of cCancer and the Mycoses Study Group Education and Research Consortium. Clin Infect Dis 2020;71:1367–1376. 31802125 10.1093/cid/ciz1008 PMC7486838

[16] Miller R , Assi M , and the AST Infectious Diseases Community of Practice. Endemic fungal infections in solid organ transplantation. Am J Transplant 2013;13:250–261. 23465018 10.1111/ajt.12117

[17] Alberti C , Bouakline A , Ribaud P , et al. Relationship between environmental fungal contamination and the incidence of invasive aspergillosis in hematology patients. J Hosp Infect 2001;48:198–206. 11439007 10.1053/jhin.2001.0998

[18] Gheith S , Ranque S , Bannour W , et al. Hospital environmental fungal contamination and aspergillosis risk in acute leukaemia patients in Sousse (Tunisia). Mycoses 2015;58:337–342. 25809008 10.1111/myc.12320

[19] Cohn SM , Pokala HR , Siegel JD , et al. Application of a standardized screening protocol for diagnosis of invasive mold infections in children with hematologic malignancies. Support Care Cancer 2016;24:5025–5033. 27518197 10.1007/s00520-016-3367-z

[20] Hopkins CC , Weber DJ , Rubin RH. Invasive aspergillus infection: possible non-ward common source within the hospital environment. J Hosp Infect 1989;13:19–25. 2564014 10.1016/0195-6701(89)90091-1

[21] Pokala HR , Leonard D , Cox J , et al. Association of hospital construction with the development of healthcare associated environmental mold infections (HAEMI) in pediatric patients with leukemia. Pediatr Blood Cancer 2014;61:276–280. 23970381 10.1002/pbc.24685 PMC4048739

[22] National Healthcare Safety Network. Patient Safety Component Manual. Centers for Disease Control and Prevention website. https://www.cdc.gov/nhsn/pdfs/pscmanual/pcsmanual_current.pdf. Published 2025. Accessed February 20, 2025.

