# Supplementary material for: Multiyear environmental surveillance in a pediatric teaching hospital: association between airborne mold spores and invasive mold infections
Source: Infect Control Hosp Epidemiol. 2025 Sep 10;46(11):1119–26. doi: 10.1017/ice.2025.10264 (PMC12620064; doi:10.1017/ice.2025.10264)
Supplement: Phillips et al. supplementary material 1 — Phillips et al. supplementary material [file S0899823X2510264Xsup001.docx]

**Supplemental Materials**

**Accompanying:**

**Multiyear Environmental Surveillance in a Pediatric Teaching Hospital: Association Between Airborne Mold Spores and Invasive Mold Infections**

**Authors:** Bethany Phillips, MPH, CIC^1^; Zachary M. Most, MD, MSc^1,2^; Bryan Connors, MS, CIH^3^; Patricia Jackson, RN, MA, CIC, FAPIC^4^; Michael E. Sebert, MD^1,2^

1- Infection Prevention and Control, Children’s Health System of Texas, Dallas, TX, USA

2- Division of Infectious Diseases, Department of Pediatrics, University of Texas Southwestern Medical Center, Dallas, TX, USA

3- Environmental Health & Engineering, Newton, MA, USA

4- Infection Prevention, Scottish Rite for Children, Dallas, TX, USA

*Hospital ventilation system*

All inpatient units and operating rooms monitored as part of this work were served by HVAC systems equipped with central HEPA-filtration at the level of the air handling unit. This provided filtration for air supplied to patient rooms as well as to adjoining corridors and nursing stations. Recirculated air was mixed with fresh air before passing again through the central HEPA-filters. Point-of-use HEPA-filtration at the level of air supply to individual patient rooms was not present for any locations. Laminar air supply was likewise not utilized in any patient rooms. Air changes per hour (ACH) were verified on an annual basis and were as follows: ≥ 2 ACH for corridors and nursing stations, ≥ 6 ACH for neutral-pressure patient rooms, ≥ 12 ACH for all positive-pressure patient rooms including those on the SCT unit, ≥ 20 ACH for operating rooms.

*Additional IMI Prevention Measures*

Other routine practices intended to reduce the risk of IMI focused on management of construction and renovation, promptly addressing water leaks, environmental cleaning practices, and cleaning and handling of linens. Our hospital is an older facility with sections first built in the 1960s and has undergone numerous expansions and renovations over subsequent years. Plans for construction activities were reviewed in advance by IP, and an infection control risk assessment (ICRA) was used to determine the required containment measures for each project. Infection preventionists then conducted weekly rounds to ensure proper implementation of required measures, although systematic data on such compliance was not recorded. Containment for major construction and renovation activities generally required that the site be under negative pressure behind rigid barricades with an anteroom. Water leaks or intrusions were evaluated by the facilities engineering department and IP, but a longitudinal database of these events was not maintained. Any building materials that had not dried within 72 hours as measured by a hand-held moisture meter were removed and replaced.

Cleaning within the hospital by the environmental services department was monitored using a UV fluorescent marker system to assess post-discharge cleaning of patient rooms. For prolonged durations of hospitalization, patients were moved at least monthly if possible to facilitate deeper cleaning of the room. Linens were laundered by an external healthcare laundry service that was inspected by the IP program and certified by the TRSA^®^ association for the Linen, Uniform and Facility Services Industry. Our IP program did not perform routine fungal cultures from linens.

Severely immunocompromised patients were required to stay on their unit except for medically necessary procedures in order to reduce potential exposure to mold spores that may be present in public areas of the hospital with higher traffic. Such patients, however, were not restricted to their individual rooms and were allowed to go to the unit playroom. Prior to the COVID-19 pandemic, these patients were also instructed to wear an N95 respirator, if possible, when it was necessary to go off the unit, although fit testing was not performed. This practice ended early in the pandemic when supplies of N95 respirators became limited because its efficacy for preventing infections was unclear. These restrictions applied when the absolute neutrophil count was < 500 cells/μL and for SCT patients within 60 days (autologous SCT) or 100 days (allogeneic SCT) of transplantation. Mold-active antifungal prophylaxis was provided to patients with acute myelogenous leukemia, selected acute lymphoblastic leukemia (ALL) patients with T-cell ALL, relapsed or refractory B-cell ALL, or chromosomal disorders such as trisomy 21, and to allogeneic SCT patients within 6 months after transplant.

*Volumetric air sampling and classification of fungal isolates*

Regular air sampling for fungal cultures was conducted approximately monthly starting in November 2018 using a bioaerosol impactor and Sabouraud dextrose agar plates. AFL data are reported through October 2023, after which a modified sampling process was adopted in preparation for demolition of an adjoining facility. Two volumetric samples were collected monthly from 1000 L of air taken approximately 1 m above the floor in common areas such as hallways and nursing stations on each of the eleven units listed. Sampling locations on each unit were well separated from each other. Samples were not collected within occupied patient rooms. Samples were also collected through December 2020 on two additional HEPA-filtered units that intermittently accommodated overflow PICU patients. Starting in January 2021, monthly samples were additionally taken in the main and cardiac operating rooms (OR). Samples from external air were collected immediately outside the hospital after completion of indoor testing.

The decision to collect samples for AFL measurement in corridors and at nursing stations rather than in patient rooms was made for several reasons. Because our facility does not have point-of-use HEPA-filtration for the air supply to patient rooms, air sampled on the unit but outside rooms was expected to be representative of the overall air supplied to the unit by the HVAC system. Unoccupied patient rooms are frequently not available due to high patient census. Sampling in unoccupied rooms would often have also required working around the environmental services and nursing teams while cleaning and preparing the room for the next patient, with concern that these activities could also impact the measured AFL. We elected not to sample in occupied rooms to minimize disruption to patients. Two samples per unit were collected so as to have multiple measurements for assessing reproducibility of results while minimizing the total number of samples in consideration of the time and expense required for this activity.

Volumetric air sampling using a bioaerosol impactor that deposits airborne material from a fixed volume of air onto an agar plate for culture was employed for this work. This method has been endorsed in the CDC HICPAC guidelines on environmental infection control as suitable for sampling air for fungal spores.^1^ In contrast, settle plates—which measure spores passively settling on agar plates over a fixed duration of time—are not recommended for this purpose because some spores may remain suspended in the air indefinitely. We opted not to use spore count methods that rely on morphologic identification of spore types by microscopy independent of culture. Although these methods may provide faster turnaround times, the results do not differentiate viable from non-viable fungal spores and have been considered more appropriate for investigation of environmental mold allergy than for questions related to infection.^2^ Furthermore, direct microscopic examination of fungal spores in the absence of culture is unable to differentiate some clinically important pathogens (*Aspergillus* versus *Penicillium*, for instance, for which the conidia are not distinguishable by light microscopy).^3^

Testing initially employed a single-stage Andersen-type Zefon A6 impactor (Zefon International, Ocala, FL, USA) but switched in January 2019 to using a SAS Super 180 air sampler (BioScience International, Rockville, MD, USA). The SAS device sampled 1000 L air for each plate, whereas the Zefon A6 impactor used during the first two months of the project was less precise but also sampled approximately 1000 L air. Samples without fungal detection were treated as having zero CFU/m^3^. The limit of detection (LOD) for an individual sample was therefore approximately 1.0 CFU/m^3^. With two such samples being taken from each unit on a given day, non-zero values as low as 0.5 CFU/m^3^ could be obtained when averaged for the unit. When AFL was averaged over several units representing a larger service line or calculated on a facility-wide basis, the averaged values could be proportionately even smaller when compared to the LOD for a single sample.

Plates for environmental fungal samples were sent to Aerobiology Laboratory Associates, Inc. (Golden, CO, USA) for quantitative culture and identification using incubation at 25°C for 5 days. Identification for all fungal isolates was based on morphology rather than molecular methods such as MALDI-TOF MS (matrix-assisted laser desorption/ionization time-of-flight mass spectrometry). As a result, organisms that did not sporulate under the selected culture conditions were listed simply as non-sporulating isolates. Additional incubation at 37°C or subculturing to different media to promote sporulation was not performed. Whole-genome sequencing was not feasible due to the retrospective nature of analysis for this project during which environmental fungal isolates were not preserved.

Fungal isolates identified by AFL monitoring were classified as opportunistic fungal pathogens or as those considered non-pathogens for surveillance purposes. Although a wide range of fungal species have been reported to cause human disease on at least some occasions, the objective of making this distinction was to differentiate AFL attributable to organisms such as *Cladosporium* (frequently found in air samples but very infrequent causes of infection) from others that are common pathogens among immunocompromised patients.

In addition to *Aspergillus* spp., other opportunistic fungal pathogens identified through AFL monitoring included *Penicillium*, *Fusarium*, *Bipolaris* or *Curvularia* (for which morphologic identification is ambiguous and which are therefore considered together for this work), *Alternaria*, *Mucor*, and *Rhizopus*, as well as infrequently *Aureobasidium*, *Paecilomyces* or *Scopulariopsis*. *Aspergillus* teleomorphs such as *Eurotium* were included with total *Aspergillus* counts. Non-pathogens included *Cladosporium*, as well as infrequently *Chrysosporium*, *Nigrospora*, or *Epicoccum*. Non-sporulating isolates were also tabulated with the non-pathogens, although these fungi could not be further identified, and some could have represented opportunistic pathogens. Yeast and yeast-like molds such as *Geotrichum* were also occasionally reported. These organisms were retained within the counts for total AFL as a reflection of overall air quality but were not included in opportunistic pathogen counts because patient infections with yeast were excluded from IMI surveillance.

*Surveillance methods for IMI*

Systematic IMI surveillance was conducted by the IP program throughout the study period. Potential IMI cases were primarily identified based on direct reporting to IP by the microbiology laboratory and pathology department of all positive fungal cultures or histopathology showing invasive fungal elements. Reports from clinicians of suspected IMI cases were also evaluated in the absence of positive cultures or histopathology, but such reporting was not systematic and likely incomplete. An automated report of fungal cultures through the electronic medical record was evaluated but not utilized because results captured in this way were substantially delayed compared to the direct reporting process already in place. Patient charts were reviewed for each potential IMI case to assess whether EORTC/MSG criteria^4^ for proven, probable or possible IMI were satisfied. For cases meeting these criteria, the HO or CO status was evaluated based on the criteria in Table 1. These locally-developed definitions for healthcare-associated events have been useful during discussions with clinical teams to acknowledge uncertainty around the incubation period of IMI while still capturing infections for surveillance and monitoring trends.

Initial case evaluations conducted by infection preventionists were validated through review by the IP medical director or associate medical director. Although *Aspergillus* galactomannan (AGM) results were included in chart reviews when available, positive AGM results were not systematically captured as independent triggers for review. Likewise, text from radiology reports and clinician notes was not systematically mined to identify potential cases. The emphasis on positive fungal cultures and histopathology for our surveillance likely contributed to the high frequency of proven IMI cases relative to probable and possible IMI.

*Initial framework for interpreting and managing AFL results*

The initial framework used by our IP program for interpreting AFL results considered both total and opportunistic fungal pathogen AFL. After classifying and tabulating results, the total and opportunistic fungal pathogen AFL values were employed to assign each unit into an action category based on the thresholds in Table 2. The higher category was used if results from total and opportunistic fungal pathogen counts were in different levels. Category assignments were based on the averages of the two AFL samples taken for each unit on a given date. Units classified as “green” based on the results received were deemed to be within an acceptable range, therefore no further action was required by IP. Units falling within the “yellow” or “red” action categories, however, were investigated further by the IP program.

For units in the “yellow” category, the investigation would include a visual inspection for any signs of water damage, dust, or outside air intrusion; evaluation of any local construction projects; inquiries with the engineering facility team about any recent work or maintenance including issues with the HVAC system or leaks reported on the unit; and discussion with unit-based staff. Any issues identified, such as dust or leaks, would be addressed by the appropriate team(s). No additional air cultures would be required in response to the “yellow” category.

Units in the “red” category would receive prompt investigation and action from the IP program. All the actions described for the “yellow” category would be completed, plus a visual inspection by the engineering department of the unit’s HVAC and HEPA filtration system. Once any issues were resolved, repeat air cultures would be collected on the unit. If repeat cultures fell within a lower range (“green” or “yellow”), no further action would be needed, and frequency of cultures would return to the regular monthly schedule. If the results remained in the “red” category, more detailed inspections were conducted by a multidisciplinary team including the infection preventionists, IP medical director(s), engineering facility staff, environmental services staff, and unit-based leadership. Provision of antifungal prophylaxis and relocation of patients were not part of the routine approach to “red” category AFL results. Implementation of such exceptional measures on two occasions—described in the discussion section of the main text—was recommended in response to particularly high AFL measurements with a predominance of pathogenic fungal isolates (*Aspergillus* and/or *Penicillium* species).

It is important to note that the initial framework for characterizing the air sampling data described above was developed to guide the priority and type of follow-up actions during and specific to the HVAC system maintenance project. Any single or small number of sample value(s) were not meant to imply a specific or known risk of patients developing an IMI. After completion of the maintenance project, the same framework was employed to guide routine IP responses to continued AFL surveillance in the absence of any other established standards. Prior to the adoption of routine AFL monitoring in late 2018, air sampling for fungal cultures had been performed at our facility on an ad hoc basis during investigations of healthcare-associated IMI.

*Consideration of AFL thresholds based on data from this study*

Our study provides data that could start to inform selection of action thresholds for future AFL monitoring in healthcare facilities. Analysis of the association between average AFL and IMI rates required pooling data across multiple units for both IMI cases and AFL monitoring because patients moved between units and because uncertainty around incubation periods made it difficult to attribute infections to precise exposure windows or locations. Prospective implementation of an AFL monitoring program, however, requires interpreting and managing new results that are reported at the level of specific inpatient units.

We therefore examined the relationship between the average AFL (“service average”) across the units providing care for hematology/oncology patients (i.e., 1 SCT unit, 3 other inpatient hematology/oncology units, and 2 PICUs) and the highest AFL measured for any of these specific units (“local unit maximum”) for that date (Figure S3). These values for individual units were themselves derived from two AFL culture plates, each representing 1000 L of air sampled that date.

Opportunistic fungal pathogen thresholds. The IMI rate appeared to increase gradually with the service average AFL for opportunistic fungal pathogens, with the highest infection rates seen when the service average AFL was greater than 2 CFU/m^3^ (Figure 2A). A service average AFL that was less than 2 CFU/m^3^ corresponded to a maximum local unit value of less than 5 CFU/m^3^ (Figure S3A). This level was higher than 98% of measurements for single inpatient units and therefore might be used as a threshold for high-level alerts that would not be expected to be triggered frequently during routine environmental surveillance.

Individual patients, however, are exposed to AFL on their local units rather than to a calculated average across units. Consideration of a lower alert threshold could therefore be reasonable as a precautionary measure. AFL values for opportunistic fungal pathogens that are ≥ 2 CFU/m^3^ on a single unit might confer elevated risk of IMI for patients with sustained exposure in that location. If interventions or other environmental fluctuations brought the AFL value down for the next measurement, however, AFL exposure averaged over the month on the unit would not be as high. A threshold of ≥ 2 CFU/m^3^ for opportunistic fungal pathogens on an individual unit could therefore be viewed as having the potential to increase the local IMI rate if not corrected. This level was higher than 91% of AFL measurements for single inpatient units and would therefore be expected to trigger alerts with a regular frequency in an environmental surveillance program such as ours that monitors more than 10 units per month.

It is also possible that higher airborne levels of opportunistic fungal pathogens occur briefly due to transient events (“bursts”) that may also increase IMI risk but may not be routinely captured by periodic air sampling.^5,6^ Given this consideration, it may be valuable to establish a low AFL threshold for follow-up actions when designing a routine air sampling program. Even small elevations in measured AFL might indicate conditions or events that have the potential if not remediated to increase the likelihood of bursts that would not themselves be evident with periodic sampling.

Because IMI cases were observed even in months when the opportunistic fungal pathogen AFL was low, these data cannot define any exposure level as being completely safe, especially for immunocompromised patients. It has been recognized, however, that complete elimination of fungi in the air and environment is not technologically feasible in occupied patient care areas even with measures such as positive pressure ventilation and HEPA-filtration^5^ without dramatically changing the way patient care is delivered to mimic a cleanroom-type environment. An appropriate goal therefore may be to identify an AFL at which the IMI risk is low even if not zero. In our data set, an average AFL value for a single unit of ≤ 1 CFU/m^3^ for opportunistic fungal pathogens corresponded to an overall service average AFL of ≤ 0.3 CFU/m^3^. Viewed at either the level of the single unit or the overall service, these AFL values therefore appeared to be associated with lower rates of IMI. 70% of opportunistic fungal pathogen AFL measurements for single inpatient units (averaged over two samples) were less than 1 CFU/m^3^.

Similar to what our IP program has previously done, an approach utilizing separate “attention” and “high-alert” threshold levels could balance the need to identify the highest-risk environmental conditions while also flagging results that may be associated with a lower degree of IMI risk. Based on the data discussed above, an “attention” threshold might be established for individual units at an opportunistic fungal pathogen AFL value of ≥ 1 CFU/m^3^, representing values that do not fall in the lowest risk range. In contrast, “high-alert” thresholds might be set at opportunistic fungal pathogen AFL values of ≥ 2-5 CFU/m^3^. Further work will be required to refine such AFL monitoring thresholds considering the variability in measurements expected between different facilities.

*Aspergillus* thresholds. Our initial framework for managing and interpreting AFL results did not include specific criteria for levels of *Aspergillus* spp. The strength of the association observed between the AFL for these particular fungi and the hematology/oncology IMI risk, however, suggests that incorporation of such criteria may be valuable. Setting specific surveillance thresholds for *Aspergillus* spp. may be challenging because there appears to be a continuum of increasing IMI risk associated with *Aspergillus* AFL even below the limit of detection for a single sample of 1 CFU/m^3^ (Figure 2B). The lowest IMI risk appeared to correspond to an *Aspergillus* AFL < 0.1 CFU/m^3^ averaged across the service units over the course of the month. It would be difficult to ascertain with confidence whether the AFL on an individual unit is below that level for any particular measurement date with a surveillance program such as ours that collected two samples per unit each from 1000 L (i.e., 1 m^3^) of air. *Aspergillus* AFL values for individual units that were ≥ 1 CFU/m^3^ resulted in service average AFL values ≥ 0.1 CFU/m^3^ (Supplemental Figure S3B) and might therefore be used as an alert threshold. These values represented 5% of such measurements for individual hematology/oncology units. Collection of more or larger volume surveillance samples would also improve sensitivity for detection of low concentrations of airborne *Aspergillus* but would require greater resource allocation.

*Additional sensitivity analyses: exclusion of AFL spike from late 2019*

A sharp increase in fungal pathogen AFL levels was seen in November and December 2019, which was substantially higher than measurements over the rest of the study period. The highest levels during these two months were recorded in the CICU and prompted relocation of patients from that unit and the provision of antifungal prophylaxis to high-risk patients (as detailed in the main text). Because these special interventions may have prevented infections and thereby affected the relationship between AFL and IMI rates, we conducted additional sensitivity analyses re-examining the association between these variables after excluding data from November and December 2019.

In these sensitivity analyses, there was still no significant association between the average monthly local cardiac unit AFL and the IMI rate among cardiac patients (0.78-fold change in IMI rate for an increase of 1 CFU/m^3^ in pathogen AFL, 95% CI 0.11-5.68, *P* = .80). For hematology/oncology patients, the association between average monthly local unit AFL and IMI rate was also largely unchanged (1.45-fold change in IMI rate for an increase of 1 CFU/m^3^ in pathogen AFL, 95% CI 0.98-2.15, *P* = .06). Exclusion of these two months, however, resulted in a newly significant association between the average monthly facility-wide AFL and the overall facility-wide IMI rate (2.23-fold change in IMI rate for an increase of 1 CFU/m3 in pathogen AFL, 95% CI 1.27-3.91, *P* = .005). Likewise, an association emerged between the average monthly facility-wide AFL and the hematology/oncology IMI rate (2.76-fold change in IMI rate for an increase of 1 CFU/m^3^ in pathogen AFL, 95% CI 1.45-5.25, *P* = .002). These patterns may have been obscured in the primary analyses by the large AFL spike in the CICU at the end of 2019, which carried over into the facility-wide data. These findings, however, should be interpreted as exploratory analyses.

*IMI rates during the COVID-19 pandemic*

The COVID-19 pandemic triggered widespread changes in masking behaviors within and outside hospitals, environmental cleaning and disinfection practices, and healthcare utilization patterns.^7-9^ Examination of the first year of the pandemic (March 2020 to February 2021) compared to the rest of the study period using interrupted time series (ITS) analysis did not show significant changes in the slope or intercept for the monthly IMI rate for hematology/oncology patients, although confidence intervals were wide (Figure S4). The aggregate rate of healthcare-associated probable or definite IMI, however, was lower during this initial pandemic period for hematology/oncology patients (incidence rate ratio [IRR] 0.27, 95% CI 0.07-1.13, *P* = .07) but not cardiac (IRR 1.43, 95% CI 0.30-6.75, *P* = .65) or other patients (IRR 1.59, 95% CI 0.34-7.38, *P* = .55).

Facility-wide AFL measurements were lower during the first year of the pandemic (median monthly value 0.52 CFU/m^3^ [IQR 0.29-0.64]) than at other times (median monthly value 0.73 CFU/m^3^ [IQR 0.46-0.99], *P* = .02). Notably however, AFL measurements on units where hematology/oncology patients received care were not lower during this period (median monthly values 0.57 [IQR 0.29-0.92] vs 0.57 [IQR 0.43-0.97] CFU/m^3^, *P* = .42).

This early pandemic reduction in AFL that did not extend to hematology/oncology units may relate to the level of activity on different units as reflected by patient census. Facility-wide patient census decreased during the first year of the pandemic (median patient-days/month 7029 [IQR 6411-7272] versus 9311 [IQR 8703-9567], *P* < .0001). Likewise, patient census decreased on the cardiac service (median patient-days/month 1099 [IQR 1044-1224] versus 1324 [IQR 1218-1387], *P* = .0001). The patient census on units caring for hematology/oncology patients (including PICU), however, was unchanged during this period (median patient-days/month 1010 [IQR 941-1042] versus 1026 [IQR 992-1079], *P* = .21), likely due to the inability to delay important care needed by this patient population.

The reason for this apparent reduction in the hematology/oncology IMI rate is unknown. Considering that the AFL on units caring for these patients was not significantly different during this time, it is plausible that increased masking related to the pandemic by these patients while remaining on their units may have contributed by decreasing effective exposure to an AFL that was itself unchanged. Although patient census was not significantly decreased on the hematology/oncology units during this period of the pandemic, changes in staff and visitor traffic on the units related to pandemic restrictions may have also reduced exposure to mold spores carried into the facility on persons or their belongings (but without these additional spores becoming airborne and contributing to the AFL, which was unchanged). Other behavioral changes during the pandemic such as increased time spent in private rooms rather than common spaces may also have impacted the IMI risk. Data are not available, however, regarding these behavioral and human traffic patterns.

*Correlations between AFL measurements for different fungal categories*

We examined the extent to which AFL values for all fungi, opportunistic fungal pathogens, and *Aspergillus* spp. were interrelated. Correlations among these categories of organisms are shown in Supplemental Figure S5 for average monthly AFL values across the units caring for hematology/oncology patients. Opportunistic fungal pathogen AFL demonstrated a moderate correlation with total fungal AFL (Pearson correlation coefficient r = 0.60, 95% CI 0.41-0.74). Likewise, *Aspergillus* AFL had a moderate correlation with that of opportunistic fungal pathogens (r = 0.56, 95% CI 0.36-0.71). *Aspergillus* AFL was not significantly correlated with total fungal AFL (r = 0.20, 95% CI -0.06-0.43).

*Relationship between AFL and hospital census*

Because AFL measurements were taken on occupied patient units during routine clinical operations, it was expected that results might be affected by the volume of clinical activity. This potential relationship was examined using monthly facility-wide patient days as a proxy for intensity of clinical activity. Analysis used linear regression with log-transformed values for both monthly average facility-wide opportunistic pathogen AFL and patient days. A pattern of increased AFL during months with higher patient-day censuses was observed as shown in Figure S6. On average, AFL was predicted to increase by 2.4-fold (95% CI 1.1-5.3, *P* = .03) in association with a doubling of the monthly inpatient census. This finding emphasizes the impact that routine clinical activity can have on AFL beyond that of other factors such as ventilation systems and construction.

*Impact of SCT unit engineering controls on AFL*

The 12-bed SCT unit at our hospital is designed to provide protective isolation with positive pressure ventilation for all patient rooms. As with the other inpatient units sampled during this study, the SCT unit is equipped with central HEPA-filtration. The unit as a whole is also at positive pressure relative to the adjoining areas of the building. Access to the SCT unit requires going through paired doors that open sequentially to maintain the pressure differential. We examined our environmental surveillance data to assess what impact these engineering controls had on AFL by comparing measurements for this unit with those for an adjoining hematology/oncology unit on the other side of the same floor of the same building tower, also with central HEPA-filtration but without these other design features. Only one patient room out of twelve on the adjoining unit is under positive pressure. Total fungal AFL was lower on the SCT unit (median 1.0 CFU/m^3^, IQR 0.5-2.0) as compared to the adjoining unit (median 2.0 CFU/m^3^, IQR 1.0-2.5; *P* = .04 by Wilcoxon matched-pairs signed rank test). AFL for fungal pathogens also showed a trend in the same direction (SCT median 0.0 CFU/m3, IQR 0.0-0.5 vs adjoining unit median 0.5 CFU/m3, IQR 0.0-0.5; *P* = .07). No significant difference was observed for *Aspergillus* AFL (median 0.0 CFU/m3 and IQR 0.0-0.0 for both units; *P* = .68), although the number of non-zero paired measurements for comparison was small. These observations suggest that the protective isolation engineering controls may be effective in reducing AFL. We cannot, however, exclude the possibility that the AFL differences observed may have resulted at least in part from differences between the units in terms of occupancy or traffic, including possible behavioral impacts of restricted access to the SCT unit.

*Outdoor AFL measurements and IMI rates*

Although outdoor AFL measurements did not show associations with healthcare-associated IMI rates comparable to those seen with AFL measured on local units, several patterns that were observed should be considered. The *Aspergillus* outdoor AFL was slightly but significantly associated with the facility-wide IMI rate (1.14-fold increase for a 1 CFU/m^3^ increase in AFL [95% CI 1.02-1.27; *P* = .02]). When broken down by service line, however, the *Aspergillus* outdoor AFL was not associated with the hematology/oncology IMI rate (1.03-fold increase for a 1 CFU/m^3^ increase in AFL [95% CI 0.89-1.20; *P* = .70]) but was rather associated with the cardiac unit IMI rate (1.44-fold increase for a 1 CFU/m^3^ increase in AFL [95% CI 1.09-1.88; *P* = .009]). This finding was unexpected because the cardiac service IMI rate was not associated with the indoor *Aspergillus* AFL measured on the local units. It is uncertain whether this represents a causal association, but it is possible that spores from the outdoor environment carried into the hospital on clothing or belongings of staff and/or visitors occasionally result in wound infections in this patient population through direct inoculation rather than an airborne route.

A small, protective association was also observed between the non-*Aspergillus* fungal pathogen AFL in outdoor air and the hematology/oncology IMI rate (0.88-fold change in IMI rate for a 1 CFU/m^3^ increase in AFL [95% CI 0.78-0.99; *P* = .04]). This association became slightly stronger when a combined IMI rate was calculated for the month of the AFL measurement plus the following month (0.87-fold change in IMI rate for a 1 CFU/m^3^ increase in AFL [95% CI 0.80-0.95; *P* = .003]). This pattern was opposite the increase in IMI rate seen when higher non-*Aspergillus* fungal pathogen AFL was measured on local inpatient units. If causal, a mechanism for this association would not be evident.

**Supplemental References**

**1.** Healthcare Infection Control Practices Advisory Committee. Guidelines for Environmental Infection Control in Health-Care Facilities (2003), Updated: July 2019. Centers for Disease Control and Prevention website. https://www.cdc.gov/infection-control/hcp/environmental-control/index.html. Published 2019. Accessed February 20, 2025.

**2.** Morris G, Kokki MH, Anderson K, Richardson MD. Sampling of *Aspergillus* spores in air. *J Hosp Infect* 2000;44:81-92.

**3.** De Linares C, Navarro D, Puigdemunt R, Belmonte J. *Aspergillus* conidia and allergens in outdoor environment: a health hazard? *J Fungi (Basel)* 2023;9:624.

**4.** Donnelly JP, Chen SC, Kauffman CA, et al. Revision and update of the consensus definitions of invasive fungal disease from the European Organization for Research and Treatment of Cancer and the Mycoses Study Group Education and Research Consortium. *Clin Infect Dis* 2020;71:1367-1376.

**5.** Falvey DG, Streifel AJ. Ten-year air sample analysis of *Aspergillus* prevalence in a university hospital. *J Hosp Infect* 2007;67:35-41.

**6.** Rupp ME, Iwen PC, Tyner LK, Marion N, Reed E, Anderson JR. Routine sampling of air for fungi does not predict risk of invasive aspergillosis in immunocompromised patients. *J Hosp Infect* 2008;68:270-271.

**7.** Moynihan R, Sanders S, Michaleff ZA, et al. Impact of COVID-19 pandemic on utilisation of healthcare services: a systematic review. *BMJ Open* 2021;11:e045343.

**8.** Cash-Goldwasser S, Reingold AL, Luby SP, Jackson LA, Frieden TR. Masks during pandemics caused by respiratory pathogens—Evidence and implications for action. *JAMA Netw Open* 2023;6:e2339443.

**9.** Zhao YH, Qu H, Wang Y, et al. Detection of microorganisms in hospital air before and during the SARS-CoV-2 pandemic. *Eur Rev Med Pharmacol Sci* 2022;26:1020-1027.

**Supplemental Table S1.** Characteristics of patients with IMI by service.

| IMI case characteristics | All IMI cases  (n = 96) | Hematology/  Oncology  (n = 49) | Cardiac Units  (n = 13) | Other Services  (n = 34) |
| --- | --- | --- | --- | --- |
| Age, years (median, IQR) | 11.1 (6.2-15.2) | 10.0 (5.9-15.0) | 6.3 (0.4-13.7) | 13.8 (8.0-16.6) |
| IMI category (%) |  |  |  |  |
| Proven IMI | 74 (77) | 34 (69) | 12 (92) | 28 (82) |
| Probable IMI | 15 (16) | 8 (16) | 1 (8) | 6 (18) |
| Possible IMI | 7 (7) | 7 (14) | 0 | 0 |
| Onset category (%) |  |  |  |  |
| Definite HO | 15 (16) | 6 (12) | 7 (54) | 2 (6) |
| Probable HO | 10 (10) | 8 (16) | 1 (8) | 1 (3) |
| Possible HO | 43 (45) | 33 (67) | 2 (15) | 8 (24) |
| Community onset | 28 (29) | 2 (4) | 3 (23) | 23 (68) |
| HD of sign/symptom onset (median, IQR) | 1 (<1 to 14) | 9 (<1 to 16.5) | 15 (5.5 to 50) | <1 (<1 to 1) |
| Infection site(s) (%) |  |  |  |  |
| Pulmonary or pleural | 35 (36) | 20 (41) | 3 (23) | 12 (35) |
| Sinonasal | 20 (21) | 19 (39) | 0 | 1 (3) |
| Skin or soft tissue | 20 (21) | 8 (16) | 2 (15) | 10 (29) |
| Mediastinum or sternum | 7 (7) | 0 | 7 (54) | 0 |
| Bloodstream or disseminated | 6 (6) | 2 (4) | 0 | 4 (12) |
| Musculoskeletal | 6 (6) | 0 | 0 | 6 (18) |
| Abdomen or pelvis | 4 (4) | 1 (2) | 0 | 3 (9) |
| Lymph node | 3 (3) | 2 (4) | 1 (8) | 0 |
| Central nervous system | 3 (3) | 0 | 0 | 3 (9) |
| Endocarditis | 2 (2) | 0 | 2 (15) | 0 |
| Eye | 1 (1) | 0 | 0 | 1 (3) |
| Clinically considered contaminant or colonization rather than infection (%) | 13 (14) | 0 | 0 | 13 (38) |
| Fungal pathogen(s) (%) |  |  |  |  |
| Hyaline molds |  |  |  |  |
| Aspergillus^*^ | 31 (32) | 15 (31) | 6 (46) | 10 (29) |
| Only positive AGM | 5 (5) | 4 (8) | 1 (8) | 0 |
| Fusarium | 9 (9) | 6 (12) | 0 | 3 (9) |
| Penicillium | 4 (4) | 0 | 0 | 4 (12) |
| Paecilomyces | 1 (1) | 0 | 0 | 1 (3) |
| Trichoderma | 1 (1) | 0 | 0 | 1 (3) |
| Hyaline mold, NOS | 3^†^ (3) | 1^†^ (2) | 0 | 2 (6) |
| Dematiaceous molds |  |  |  |  |
| Bipolaris/Curvularia | 21 (22) | 13 (27) | 5 (38) | 3 (9) |
| Alternaria | 5 (5) | 5 (10) | 0 | 0 |
| Exserohilum | 4 (4) | 3 (6) | 1 (8) | 0 |
| Exophiala | 2 (2) | 1 (2) | 0 | 1 (3) |
| Cladosporium | 4 (4) | 1 (2) | 0 | 3 (9) |
| Dematiaceous mold, NOS | 3 (3) | 1 (2) | 0 | 2 (6) |
| Mucorales^‡^ |  |  |  |  |
| Rhizopus | 6 (6) | 5 (10) | 0 | 1 (3) |
| Rhizomucor | 2 (2) | 0 | 1 (8) | 1 (3) |
| Mucor | 2 (2) | 0 | 0 | 2 (6) |
| Zygomycetes, NOS | 2 (2) | 2 (4) | 0 | 0 |
| Dimorphic fungi |  |  |  |  |
| Histoplasma | 4 (4) | 1 (2) | 1 (8) | 2 (6) |
| Coccidioides | 1 (1) | 0 | 0 | 1 (3) |
| Trichophyton | 2 (2) | 0 | 0 | 2 (6) |
| Nannizziopsis | 1 (1) | 0 | 0 | 1 (3) |
| No fungal pathogen identified | 7 (7) | 7 (14) | 0 | 0 |
| Multiple mold species^§^ | 15 (16) | 9 (18) | 1 (8) | 5 (15) |

IMI, invasive mold infection; IQR, interquartile range; HO, hospital-onset; HD, hospital day; AGM, *Aspergillus* galactomannan; NOS, not otherwise specified

^*^Including cases with positive AGM testing only

^†^Including one isolate identified as *Penicillium*/*Paecilomyces*

^‡^Including isolates identified only as Zygomycetes, which could represent species other than Mucorales.

^§^Isolates from cases with multiple mold species are also listed separately with the individual molds identified

**Supplemental Table S2.** Types of fungi sampled from air and associations between AFL measurements and IMI rates.

| Healthcare-associated proven and probable IMI cases per 10,000 patient days | Change in IMI rate per change of 1 CFU/m^3^ in average monthly fungal pathogen AFL | | | | | | | |
| --- | --- | --- | --- | --- | --- | --- | --- | --- |
|  | Total fungal AFL | | Fungal pathogen AFL | | *Aspergillus* AFL | | Non-*Aspergillus* fungal pathogen AFL | |
|  | Fold change  (95% CI) | *P* value | Fold change  (95% CI) | *P* value | Fold change (95% CI) | *P* value | Fold change (95% CI) | *P* value |
|  | Facility-wide AFL | | | | | | | |
| Facility-wide rate |  |  |  |  |  |  |  |  |
| Same month as AFL | 1.13  (0.95-1.33) | .17 | 1.19  (0.94-1.51) | .16 | 2.19  (0.95-5.04) | .07 | 1.23  (0.88-1.71) | .22 |
| Same and following month as AFL | 1.10  (0.98-1.25) | .12 | 1.14  (0.94-1.36) | .18 | 1.68  (0.86-3.31) | .13 | 1.17  (0.91-1.50) | .21 |
|  | Local Unit AFL | | | | | | | |
| Hematology/Oncology rate |  |  |  |  |  |  |  |  |
| Same month as AFL | 1.08  (0.89-1.32) | .42 | 1.48  (1.00-2.19) | .05 | 15.9  (2.8-90.7) | .002 | 1.39  (0.88-2.19) | .16 |
| Same and following month as AFL | 1.10  (0.96-1.27) | .16 | 1.43  (1.08-1.90) | .01 | 5.83  (1.43-23.7) | .01 | 1.42  (1.04-1.95) | .03 |
| Cardiac units rate |  |  |  |  |  |  |  |  |
| Same month as AFL | 0.98  (0.76-1.26) | .86 | 0.72  (0.15-3.46) | .68 | 0.59  (0.05-7.01) | .67 | 0.64  (0.09-4.79) | .67 |
| Same and following month as AFL | 0.97  (0.81-1.17) | .77 | 0.69  (0.20-2.32) | .54 | 0.57  (0.09-3.5) | .55 | 0.61  (0.13-2.83) | .53 |
|  | Outdoor AFL | | | | | | | |
| Facility-wide rate |  |  |  |  |  |  |  |  |
| Same month as AFL | 1.01  (1.00-1.01) | .15 | 0.99  (0.94-1.05) | .75 | 1.14  (1.02-1.27) | .02 | 0.94  (0.87-1.02) | .12 |
| Same and following month as AFL | 1.00  (1.00-1.01) | .12 | 0.99  (0.95-1.03) | .55 | 1.14  (1.06-1.24) | .0007 | 0.93  (0.88-0.99) | .02 |
| Hematology/Oncology rate |  |  |  |  |  |  |  |  |
| Same month as AFL | 1.01  (1.00-1.01) | .22 | 0.93  (0.85-1.01) | .08 | 1.03  (0.89-1.20) | .70 | 0.88  (0.78-0.99) | .04 |
| Same and following month as AFL | 1.00  (1.00-1.01) | .46 | 0.94  (0.89-1.00) | .046 | 1.09  (0.98-1.20) | .11 | 0.87  (0.80-0.95) | .003 |
| Cardiac units rate |  |  |  |  |  |  |  |  |
| Same month as AFL | 1.01  (1.00-1.03) | .057 | 1.07  (0.94-1.22) | .33 | 1.44  (1.09-1.88) | .009 | 0.96  (0.79-1.17) | .71 |
| Same and following month as AFL | 1.01  (1.00-1.02) | .03 | 1.01  (0.91-1.13) | .80 | 1.30  (1.07-1.57) | .009 | 0.90  (0.76-1.08) | .26 |

IMI, invasive mold infection; AFL, airborne fungal load; CFU, colony forming units

Notes: All rates are calculated based on combined numbers of proven and probable IMI cases. Local air pathogen counts for the hematology/oncology population include those taken on inpatient hematology/oncology floors, stem cell transplant unit, and pediatric intensive care units. Local air pathogen counts for the cardiac population were limited to those taken in the CICU as samples were not collected on the acute care cardiology floor.

**Supplemental Table S3**. Percentage of air surveillance cultures positive for different fungi.

| Fungal types identified | Percentage of Air Samples Positive^*^ | | |
| --- | --- | --- | --- |
|  | Inpatient Units  (n = 1405) | Operating Rooms  (n = 136) | Outdoor Air Samples  (n = 77) |
| Any fungal isolate(s) | 79.0 | 27.2 | 100.0 |
| Any opportunistic fungal pathogen(s) | 37.1 | 6.6 | 96.1 |
| *Aspergillus* spp. | 9.1 | 3.7 | 71.4 |
| *Penicillium* spp. | 16.0 | 0.7 | 53.2 |
| Dematiaceous molds | 13.5 | 2.9 | 20.8 |
| Mucorales | 2.0 | 0 | 26.0 |
| *Fusarium* spp. | 0.7 | 0 | 26.0 |
| *Cladosporium* spp. | 37.7 | 8.8 | 94.8 |
| Yeast^†^ | 7.8 | 2.9 | 3.9 |
| Non-sporulating fungal isolates | 47.4 | 14.0 | 61.0 |
| Other fungal species^‡^ | 6.3 | 0.7 | 10.4 |

^*^Percentages of environmental surveillance specimens each from 1000 L of air that grew one or more colonies of the listed fungal types.

^†^Includes yeast, which were not further identified, and yeast-like fungi such as *Geotrichum* spp.

^‡^Other fungal species represent infrequently identified organisms, including some considered opportunistic pathogens and others categorized as non-pathogens as described elsewhere in Supplemental Materials.

Notes: Data presented are descriptive statistics for all individual surveillance air cultures from the given areas. All inpatient units sampled were equipped with HEPA-filtration.

**Supplemental Figure Legends**

**Supplemental Figure S1.** Histograms showing frequency distributions of AFL measurements for all fungi (A, D, G), opportunistic fungal pathogens (B, E, H), and *Aspergillus* species (C, F, I) in environmental surveillance samples from inpatient units (A-C), operating rooms (D-F), and outdoor air (G-I). AFL, airborne fungal load; CFU, colony forming units

**Supplemental Figure S2.** Comparison of facility-wide average AFL values for opportunistic fungal pathogens measured on inpatient units with the AFL measured from outdoor air immediately afterwards (correlation coefficient = -0.20). Dots represent measurements for single dates. AFL, airborne fungal load; CFU, colony forming units

**Supplemental Figure S3.** AFL for (A) opportunistic fungal pathogens and (B) *Aspergillus* spp. on 6 inpatient units where hematology/oncology patients received care (1 stem cell transplant unit, 3 other hematology/oncology units, and 2 pediatric intensive care units). Average values for all units combined are compared with the maximum values for any of the individual units on a given day. The number of overlapping data points for each pair of values is indicated by the size of the dot as shown in the keys. AFL, airborne fungal load; CFU, colony forming units

**Supplemental Figure S4.** ITS analysis of monthly rates of healthcare-associated probable or proven IMI among hematology/oncology patients, comparing the initial year of the COVID-19 pandemic (March 2020 to February 2021) with the pre-pandemic (January 2018 to February 2020) and following (March 2021 to December 2023) periods. Healthcare-associated IMI cases included those classified as possible, probable, or definite hospital-onset. Dots indicate observed monthly rates. Thick lines represent predicted rates from the ITS model while 95% confidence intervals are shown by thin lines. Ventilation system extended maintenance was conducted intermittently from June 2019 to August 2020. ITS, interrupted time series; IMI, invasive mold infection

**Supplemental Figure S5.** Correlations between average monthly AFL values on units caring for hematology/oncology patients for (A) all fungi vs opportunistic fungal pathogens (Pearson correlation coefficient r = 0.60, 95% CI 0.41-0.74), and (B) opportunistic fungal pathogens vs *Aspergillus* species (r = 0.56, 95% CI 0.36-0.71). AFL, airborne fungal load; CFU, colony forming units

**Supplemental Figure S6.** Variation in average monthly facility-wide AFL on sampled inpatient units for opportunistic fungal pathogens during months with the facility-wide census of admitted patient-days as shown. Dots indicate observed monthly AFL values. The thick line represents predicted AFL values from the regression model while 95% confidence intervals are shown by thin lines. AFL, airborne fungal load; CFU, colony forming units.
